# Influence of Milk on Exhaled Carbon Monoxide (CO) Measurement by Portable CO Monitors

**DOI:** 10.1155/2022/6714601

**Published:** 2022-12-09

**Authors:** Kiho Miyoshi, Narito Kurioka, Sadahiro Kawazoe, Takashi Miyawaki

**Affiliations:** ^1^Department of Food and Nutrition, Graduate School of Home Economics, Kyoto Women's University, Kyoto 605-8501, Japan; ^2^Kyoto Association for Tobacco Control, Kyoto, Japan

## Abstract

**Background:**

A portable breath carbon monoxide (CO) monitor has a high cross-sensitivity to hydrogen (H_2_). This study examined the influences of H_2_ after consuming milk on the detected CO values using three types of portable CO monitors.

**Materials and Methods:**

Exhaled breath from seven participants (four healthy nonsmokers and three smokers with otherwise unknown comorbidities) was collected in sampling bags. The participants then consumed 200 mL of milk, and the exhaled breath of each was collected in separate bags every 30 minutes until 9 hours later. CO and H_2_ in the bag were measured using a gas chromatograph as a reference analyzer, and CO was also measured using three types of portable CO monitors.

**Results:**

After consuming milk, H_2_ levels were significantly higher, and CO levels were not significantly elevated as measured by the reference analyzer. However, CO levels in monitors A and B were significantly elevated, even though participants did not smoke. The H_2_ levels in the reference analyzer significantly increased and reached a maximum 4.5 hours after consuming milk. The difference in CO levels between the reference analyzer and each monitor increased significantly after 5 or 5.5 hours.

**Conclusions:**

This study suggested that the breath CO monitors with a cross-sensitivity to H_2_ responded to H_2_ as CO in the exhaled gas and measured higher than actual values after milk consumption. The extent of the influence of H_2_ differed depending on the type of CO monitor. It is necessary to consider milk consumption when assessing the smoking status of people using portable CO monitors.

## 1. Introduction

One of the toxic substances produced by smoking is carbon monoxide (CO), a product of converting heme to biliverdin by heme oxygenase in microsomes. CO binds to heme molecules such as hemoglobin, causing tissue hypoxia and oxidative stress [1].

An easy and objective method for evaluating smoking status is to measure the CO in smokers' breath using a CO monitor, which is the “stethoscope” of a tobacco treatment specialist. It is also used to measure the nicotine dependence level and passive smoke exposure and plays an important role in the titration of combination medication dosing. It is a powerful motivational tool for quitting smoking. Currently, three types of portable breath CO monitors are used to assess smoking status in smoking cessation programs [2–4]. It has been reported that the sensor of the CO monitors has a high cross-sensitivity to hydrogen (H_2_). In people with hypolactasia or lactose intolerance, lactose in milk, which is not metabolized in the small intestine, is changed into H_2_ by the intestinal bacteria and is absorbed. In such cases, the portable CO monitors may erroneously measure H_2_ as CO levels of the exhaled breath [5].

Rates of hypolactasia or lactose intolerance vary widely by race, but approximately 70% of the world population have hypolactasia [5, 6], more than 90% of Japanese people have decreased lactase activity, and 20% are lactose intolerant [7]. In individuals with hypolactasia or lactose intolerance, intake of milk and other dairy products containing lactose can cause gastrointestinal symptoms such as diarrhea, abdominal pain, and increased gastric emptying. However, not everyone with hypolactasia or lactose intolerance has gastrointestinal symptoms [8], and H_2_ is often detected in the breath of people who consume milk or lactose but do not have such symptoms.

In this study, to properly assess CO levels detected in the portable breath CO monitor, we examined the influence of consuming milk on the CO values using three types of monitors and compared the values with that of a reference analyzer.

## 2. Materials and Methods

### 2.1. Participants

Participants were seven individuals (four healthy nonsmokers and three smokers with otherwise unknown comorbidities) without respiratory diseases and milk allergies. Moreover, they were not diagnosed as lactose intolerant. They were either students or affiliated with a Japanese university and recruited in a class on health as volunteers between December 2019 and December 2020. Participants with obvious milk allergies and lung or bronchial abnormalities were excluded. Written informed consent was obtained from all participants.

### 2.2. Procedure

**Figure 1:**
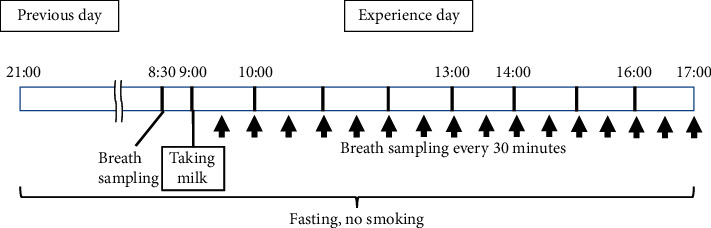
Study design.

Participants consumed no milk and dairy products the day before the study. After an overnight fast (only water intake was allowed) and nonsmoking, at 8:30 a.m., participants exhaled and inhaled completely, held their breath for 15 seconds, and then exhaled rapidly into a 600 mL sampling bag (Taiyo Corporation, Osaka, Japan) in which the exhaled air was collected. At 9 a.m., participants consumed 200 mL of milk (containing 8.6 g lactose). After consuming milk, the exhaled breath of every participant was collected in a sampling bag every 30 minutes until 5 p.m., in the same manner. Participants did not consume any food or drink and were prohibited from smoking during the study. The participants were asked about their subjective gastrointestinal symptoms to evaluate them as lactose intolerant after consuming milk. The study design is shown in Figure 1.

A gas chromatograph with a semiconductor detector (TRIlyzer mBA-3000, Taiyo Corporation, Osaka, Japan) was used as a reference analyzer. Some of the exhaled breath was injected into the reference analyzer from each sampling bag, and CO and H_2_ were measured. The remaining exhaled air in the bag was injected into three different types of portable CO monitors (monitor A: Smokerlyzer (PICOplus®), Bedfont Inc.; monitor B: Micro CO monitor, Vyaire Medical Inc.; and monitor C: Smokerlyzer (PICO advance®)). Before the study, the analyzer was calibrated with a mixture of CO and air [9]. CO values were measured by the reference analyzer and three monitors, and H_2_ values were measured by the reference analyzer. CO and H_2_ values were compared in time series.

### 2.3. Statistical Analysis

Data were analyzed using IBM SPSS version 25 (IBM Corp., NY, USA). The Wilcoxon signed-rank test was used to compare the paired groups (levels before consuming milk and at each time after consuming milk). Spearman's correlation coefficient was used to evaluate the relationship between the two parameters. Data are presented as medians (first and third quartiles). Statistical significance was set at *P* < 0.05. Significant differences were declared at *P* ≤ 0.05 and tendencies between 0.05 ≤ *P* ≥ 0.1.

The study was approved by the Research Ethics Committee of Kyoto Women's University (approval number 2019-25) and was conducted in accordance with the guidelines of the Declaration of Helsinki.

## 3. Results

Table 1 shows the characteristics of the participants and CO and H_2_ levels before and after consuming milk. Participants were aged between 22 and 60 years, five men and two women, three smokers, and four nonsmokers. Only one had occasional abdominal symptoms after consuming milk; however, the participant had no symptoms in this study.

Before consuming milk, participants' CO levels ranged from 1.0 to 16.4 ppm by the reference analyzer and from 1 to 9 ppm, 0 to 13 ppm, and 1 to 10 ppm in monitors A, B, and C, respectively. H_2_ levels in the reference analyzers ranged from 1.6 to 19.1 ppm. Participants had no abdominal symptoms after consuming milk in this study. After consuming milk, the CO levels of almost all of the participants increased compared with those before consuming milk. H_2_ levels increased 1.8- to 16.8-fold compared to those before consuming milk.

Table 2 shows CO levels measured by monitors A, B, and C and the reference analyzer and H_2_ levels measured by the reference analyzer before and after consuming milk. No significant differences were found in the median CO levels measured by the reference analyzer and monitors A, B, and C before milk consumption. After milk consumption, H_2_ levels in the reference analyzer were significantly higher (*P* = 0.018) and CO levels in the reference analyzer were not significantly elevated. However, CO levels in monitors A and B were significantly elevated (*P* = 0.039 and *P* = 0.026, respectively).

**Figure 2:**
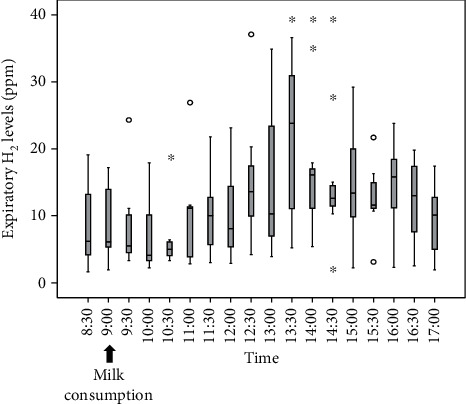
Time course of expiratory H_2_ levels. Expiratory H_2_ levels gradually increase after consuming milk and are significantly higher than those before consuming milk. ^∗^*P* < 0.05 vs. the levels before consuming milk.

Figure 2 shows the time course of H_2_ levels in the reference analyzer. The H_2_ levels in the reference analyzer significantly (*P* = 0.018) increased after consuming milk and reached a maximum at 13:30 (4.5 hours after milk intake).

**Figure 3:**
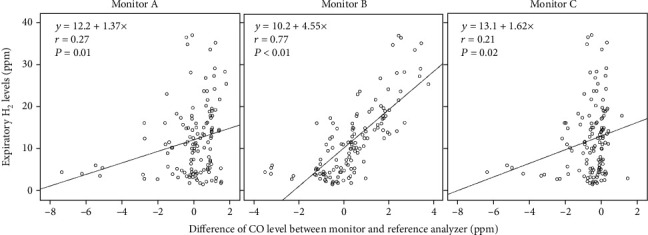
Relationship between the difference of CO level between monitors and reference analyzer and expiratory H_2_ levels measured by the reference analyzer.

Figure 3 shows the relationship between the difference in CO levels of each monitor and the reference analyzer and the expiratory H_2_ levels. The difference between the CO values of each monitor and the reference analyzer was significantly correlated with the H_2_ levels of the reference analyzer. The difference in monitor B had the strongest correlation with the H_2_ levels of the reference analyzer. In monitor B, approximately one-tenth of the hydrogen concentration in the exhaled air was mistakenly measured as CO levels.

**Figure 4:**
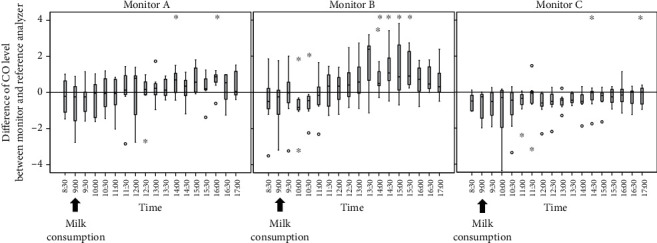
Time course of the difference in CO levels in the reference analyzer and the three monitors. ^∗^*P* < 0.05 vs. the levels before consuming milk.

Figure 4 shows the time course of the difference in CO concentration between each monitor and the reference analyzer. The difference in CO levels between the reference analyzer and each monitor showed an increasing trend after consuming milk in all monitors. In monitors A and B, it became significant at 14:00 (5 hours later). In monitor C, it became significant at 14:30 (5.5 hours later).

## 4. Discussion

This study illustrated that after consuming 200 mL of milk, the CO levels detected in monitors A and B increased significantly after 5 to 5.5 hours compared to the values before consumption, despite the fact that participants did not smoke. Individual differences were observed in the levels and duration of the increase in CO levels measured by the monitors. This is the first study to show the effect of H_2_ produced by consuming milk on the values measured by different models of portable CO monitors.

All three portable CO monitors used in this study use electrochemical gas sensors. The electrochemical analysis method measures the electric current produced in an aqueous solution by electrical oxidation by an electrode that has acted as a catalyst [10, 11]. As detailed measurement methods are not disclosed by the manufacturer, there was no consistent agreement on the CO value between models [3]. As this electrochemical sensor also reacts with H_2_, hydrogen sulfide, sulfur dioxide, nitrogen dioxide, nitrogen monoxide, and ethylene, if there is H_2_ in the expired breath, it may be erroneously measured as CO [5]. The instructions for the Bedfont Scientific Ltd. instrument describe the possibility of H_2_ crossover (interference with H_2_) but do not describe the specific effects of lactose ingestion (time and extent).

The activity of lactase, a lactose-degrading enzyme at the brush border of the small intestinal mucosal epithelium, is deficient or reduced in people with hypolactasia or lactose intolerance. Therefore, lactose, a disaccharide, is not degraded into glucose and galactose. The lactose that cannot be degraded is not absorbed in the small intestine and is fermented by intestinal bacteria in the large intestine, and H_2_ is produced. The produced H_2_ is absorbed through the intestinal mucosa, dissolved into the blood, and diffused into the alveoli via the blood circulation, and some of it is expired in the exhaled air [12]. Lactose also irritates the large intestine, causing the abdominal symptoms of lactose intolerance. The objective evaluation of lactose intolerance is done by measuring enzyme (lactase) activity using biopsy material of the small intestine [13]. In the participants of this study, a possibility of low lactase activity (subclinical lactose intolerance [14]) was suggested because of the H_2_ in their exhaled breath after consuming milk, although they were not aware of lactose intolerance. Therefore, before measuring CO levels by the breath CO monitor, it is necessary to check the consumption of milk or foods that may produce H_2_, the time after consumption, and the type of monitor used.

After consuming milk, the increase in CO levels detected in monitor C was less than in A and B. This is because the portable monitor C is the most recent model; hence, the influence of H_2_ may be minimal due to advances in technology including calibration adjustment of the monitor. However, CO monitor C might have measured a value slightly lower than the actual value.

Few studies have examined the effect of H_2_ on CO monitor readings. We have reported in a previous study that in eleven nonsmokers who consumed 400 mL of milk, the levels of the three portable CO monitors were significantly elevated from 1.5 hours to a maximum of 8 hours after ingestion, up to a maximum of 18 ppm [8]. Our previous report showed a greater degree of elevation of the CO levels compared to the present study. This is presumably because 200 mL of milk was consumed in the present study, whereas 400 mL of milk was consumed in the previous one, suggesting a dose dependence on the relationship between the amount of H_2_ produced and amount of lactose ingested. Another previous report using the Bedfont Micro Smokerlyzer monitor on four lactose-intolerant persons showed that an H_2_ concentration of 38.91 ppm in exhaled air was sufficient to record a CO level of 10 ppm, and this level is equivalent to the ingestion of 350 mL of milk [5].

There are several limitations to this study. First, various foods other than lactose produce H_2_ in the intestine, but this study focused only on lactose. Even when foods that do not require digestive enzymes, such as dietary fiber and indigestible carbohydrates, are ingested, they may pass undigested through the small intestine and be fermented by intestinal bacteria in the large intestine, producing H_2_ [15, 16]. Some participants in this study had elevated H_2_ levels before milk consumption, which might be related to the previous day's diet. Second, in the study, exhaled air was injected into the sampling bag and measured by a reference analyzer and CO monitor. Therefore, there may be a difference in levels compared to those obtained when exhaled air is directly blown into the CO monitor. Third, this study only examined a time course of up to 8 hours. Fourth, we have not been able to examine in detail the causes of H_2_ production, such as the degree of decrease in lactase activity and differences in the state of the intestinal bacteria. Fifth, the sample size of this study is small; therefore, careful consideration should be given to realistic application in clinical practice. The causes of individual differences could not be examined because of the small number of participants. Sixth, the relation between the nicotine dependence level of the smokers and their expired CO levels was not investigated. Seventh, the study lacked a control group who did not drink milk.

## 5. Conclusions

The results of this study showed that when a portable CO monitor was used to measure CO after lactose intake, the CO monitor responded to H_2_, and the measured value increased even if the exhaled air did not contain CO, regardless of whether the participant had subjective symptoms of lactose intolerance or not. The extent of the effect differed depending on the type of CO monitor. Therefore, when assessing the smoking status using portable breath CO monitors, it is necessary to consider prior consumption of milk or foods that may produce H_2_, the time after consumption, and the type of monitor used. Further studies are needed to explore the influences of foods that may produce H_2_ on the CO values using portable CO monitors.

## Figures and Tables

**Table 1:** Characteristics of the participants and H_2_ and CO levels before and after consuming milk.

	Sex	Age	Smoker or not	Habitual symptoms of lactose intolerance	Symptoms during the study	CO levels (ppm) before consuming milk				Reference H_2_ levels (ppm)	Maximum CO levels (ppm) after consuming milk				Maximum H_2_ levels after consuming milk (ppm)	Multiples compared to reference H_2_ levels
						Monitor A	Monitor B	Monitor C	Reference		Monitor A	Monitor B	Monitor C	Reference		
1	M	23	No	Sometimes	Nothing	1.0	0.0	1.0	1.2	3.8	3.0	5.0	2.0	1.6	28.4	7.5
2	M	60	No	Nothing	Nothing	3.0	2.0	2.0	2.5	1.6	4.0	4.0	2.0	2.6	26.9	16.8
3	F	22	No	Nothing	Nothing	2.0	1.0	1.0	1.2	6.2	2.0	3.0	1.0	1.2	15.0	2.4
4	F	22	No	Nothing	Nothing	2.0	3.0	1.0	1.0	19.1	2.0	4.0	2.0	1.2	35.3	1.8
5	M	27	Smoker	Nothing	Nothing	7.0	7.0	7.0	7.7	17.4	8.0	9.0	8.0	9.8	37.1	2.1
6	M	25	Smoker	Nothing	Nothing	4.0	6.0	4.0	5.5	9.0	5.0	6.0	4.0	5.4	21.7	2.4
7	M	25	Smoker	Nothing	Nothing	9.0	13.0	10.0	16.4	4.5	12.0	14.0	12.0	17.5	36.6	8.1

**Table 2:** CO levels measured by monitors A, B, and C and the reference analyzer and H_2_ levels measured by the reference analyzer before and after consuming milk.

		Before consuming milk	Maximum levels after consuming milk	*P* value
CO levels (ppm)	Monitor A	3.0 (2.0, 7.0)	4.0 (2.0, 8.0)	0.039^∗^
	Monitor B	3.0 (1.0, 7.0)	5.0 (4.0, 9.0)	0.026^∗^
	Monitor C	2.0 (1.0, 7.0)	2.0 (2.0, 8.0)	0.059
	Reference	2.5 (1.2, 7.7)	2.6 (1.2, 9.8)	0.058

Reference H_2_ levels (ppm)		6.2 (3.8, 17.4)	28.4 (21.7, 36.6)	0.018^∗^

Values are expressed in median (first quartile and third quartile). ^∗^*P* < 0.05.

## Data Availability

Data are available from the corresponding author on reasonable request.
